# Successful Recovery From a Severe Graft Dysfunction Due to Acute Rejection After Liver Transplantation: A Case Report

**DOI:** 10.7759/cureus.98504

**Published:** 2025-12-05

**Authors:** Marina Isokawa, Ryoichi Goto, Takuji Ota, Norio Kawamura, Masaaki Watanabe, Tsuyoshi Shimamura, Akinobu Taketomi

**Affiliations:** 1 Gastroenterological Surgery, Hokkaido University Graduate School of Medicine, Sapporo, JPN; 2 Department of Transplant Surgery, Hokkaido University, Sapporo, JPN; 3 Division of Organ Transplantation, Hokkaido University Hospital, Sapporo, JPN

**Keywords:** graft failure, graft rejection, hyperbilirubinemia, renal dysfunction, renal failure

## Abstract

Graft failure remains a major challenge after liver transplantation (LT), and rejection is one of its leading causes. We report a rare case of severe graft dysfunction due to acute rejection that successfully recovered without re-transplantation. A 58-year-old man with hepatocellular carcinoma (HCC) secondary to alcoholic liver cirrhosis was registered on the waiting list for deceased donor LT. He developed intractable ascites and renal dysfunction caused by hepatorenal syndrome and subsequently underwent deceased donor LT. On postoperative day 23, severe acute rejection resulted in the destruction of approximately 90% of hepatocytes in the graft and permanent renal failure. The peak total bilirubin level was 29.6 mg/dL. Although re-transplantation was considered, it was not feasible due to the severe donor shortage in Japan. Comprehensive and meticulous medical management was continued for approximately four months, and the liver graft eventually recovered, allowing the patient to return to work. This rare case demonstrates successful recovery from severe liver graft failure after transplantation through conservative treatment, and identifying clinical parameters that predict graft recovery may help determine optimal treatment strategies.

## Introduction

The development of immunosuppressive agents such as calcineurin inhibitors (CNIs) and advances in various surgical techniques and perioperative care have greatly improved clinical outcomes after liver transplantation (LT) over the past decades [1,2]. However, graft failure remains a serious complication that occurs occasionally and is an indication for re-transplantation (re-LT). Re-LT may be required in certain cases, with rates reported as 5-22% worldwide and 3% in Japan [3,4]. One of the leading causes of graft failure is rejection [3,5]. Here, we report a case of deceased donor liver transplantation (DDLT) in a patient with pre-transplant renal dysfunction, who suffered graft dysfunction with more than 90% hepatocyte destruction due to acute allograft rejection (AR), but successfully recovered without re-LT.

## Case presentation

A 58-year-old man was referred to our LT unit with alcoholic liver cirrhosis. He had previously undergone radiofrequency ablation and transcatheter arterial chemoembolization (TACE) for two hepatocellular carcinomas (HCCs) located in segments 5 and 6 five years earlier. He subsequently developed intractable ascites requiring repeated drainage and renal dysfunction due to hepatorenal syndrome. Esophageal varices were treated with endoscopic injection sclerotherapy. The patient had abstained from alcohol for 18 months before being registered on the waiting list for DDLT. Computed tomography (CT) performed for registration revealed a recurrent HCC (25 × 25 mm) in segment 4, which was treated with TACE during the waiting period. Renal function gradually worsened, and he eventually underwent DDLT 149 days after registration. The deceased donor was in his 40s. Laboratory data at the time of transplantation are shown in Tables 1-3.

**Table 1:** Complete blood count before liver transplantation Abbreviations: WBC, white blood cell; Hb, hemoglobin; Plt, platelet; PT, prothrombin time; PT-INR, international normalized ratio of prothrombin time; APTT, activated partial thromboplastin time

Parameter	Result	Unit
WBC	1900	/μl
Hb	8.4	g/dl
Plt	5.3×10⁴	/μl
PT	14.9	sec
PT-%	59.9	%
PT-INR	1.27	
APTT	53.1	sec

**Table 2:** Chemistry and serological test results before liver transplantation Abbreviations: TP, total protein; Alb, albumin; T-bil, total bilirubin; D-bil, direct bilirubin; AST, aspartate aminotransferase; ALT, alanine aminotransferase; ALP, alkaline phosphatase; γ-GTP, gamma-glutamyl transpeptidase; BUN, blood urea nitrogen; Cre, creatinine; eGFR, estimated glomerular filtration rate; CRP, C-reactive protein

Parameter	Result	Unit
TP	6.2	g/dl
Alb	2.7	g/dl
T-bil	2.4	mg/dl
D-bil	0.9	mg/dl
AST	31	IU/l
ALT	13	IU/l
ALP	245	IU/l
γ-GTP	78	IU/l
BUN	28	mg/dl
Cre	1.72	mg/dl
eGFR	33.9	ml/min
Na	137	mEq/l
K	3.9	mEq/l
Cl	102	mEq/l
CRP	1.68	mg/dl
NH₃	94	μg/dl

**Table 3:** Serological, immunological, and tumor marker tests before liver transplantation Abbreviations: TM, tumor marker; HBs-Ag, hepatitis B surface antigen; HBs-Ab, hepatitis B surface antibody; HCV-Ab, hepatitis C virus antibody; ANA, antinuclear antibody; AMA, antimitochondrial antibody; AFP, alpha-fetoprotein; DCP, des-γ-carboxy prothrombin

Parameter	Result	Unit
HBs-Ag	<0.005	IU/ml
HBs-Ab	Negative	
HCV-Ab	<1.00	S/CO
ANA	Negative	
AMA	Negative	
AFP	1.8	ng/ml
DCP	1090	mAU/ml

**Figure 1:**
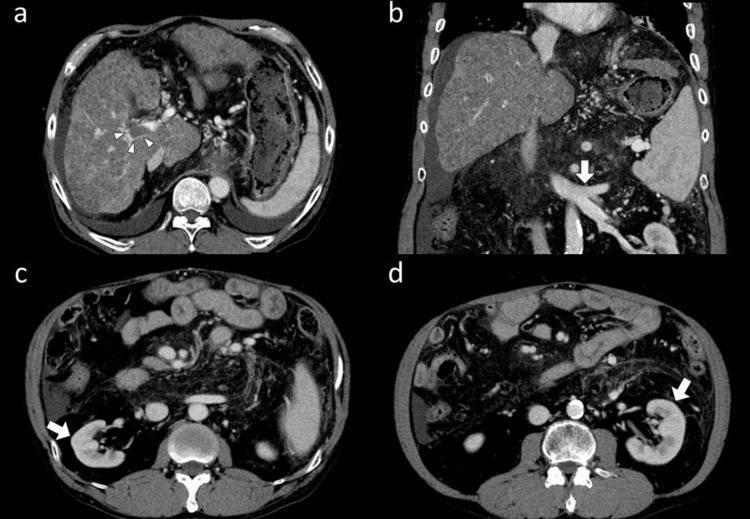
Pretransplant CT of the recipient who underwent DDLT Pretransplant CT findings of the recipient who underwent DDLT. a) The thrombus of the right portal vein was observed (arrowheads). b) Splenomegaly and splenorenal shunt (arrow) were shown. c and d) The bilateral kidneys were atrophic (arrows). Abbreviations: CT, computed tomography; DDLT, deceased donor liver transplantation

The Child-Pugh score was 12, and the Model for End-Stage Liver Disease (MELD) score was 18. Preoperative CT findings (Figure 1) demonstrated a cirrhotic liver morphology without viable tumors. A thrombus was detected in the main trunk of the right portal vein (Figure 1a). Splenomegaly, a splenorenal shunt (Figure 1b), pleural effusion, and ascites were also observed. The bilateral kidneys were atrophic (Figure 1c, 1d), suggesting pre-existing chronic kidney disease. Considering the preoperative renal dysfunction due to hepatorenal syndrome, the patient was considered to have acute-on-chronic kidney disease.

The operative time was 13 hours and 28 minutes. The cold and warm ischemia times were seven hours and 32 minutes and 34 minutes, respectively. The total blood loss was 12,110 mL.

**Figure 2:**
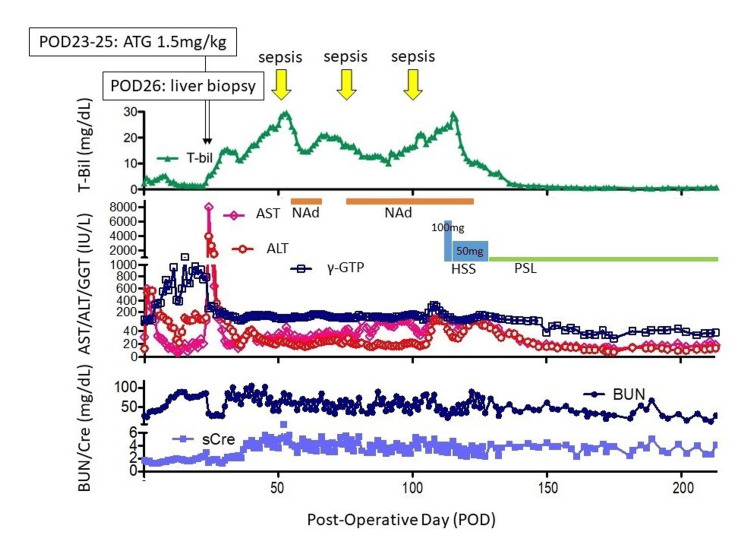
Clinical parameters post-LT The changes in values of total bilirubin (T-bil, triangles, green line) post-LT were shown in the top row. The changes in the values of aspartate aminotransferase (AST, pink empty rhombuses and line), alanine aminotransferase (ALT, red empty circles and line), and gamma-glutamyl transpeptidase (γ-GTP, blue empty squares and line) during the post-LT course were shown in middle row. Post-LT changes in values of serum creatinine (sCre, sky blue solid squares and line) and blood urea nitrogen (BUN, dark blue solid circles and line) were shown in bottom row. Permanent hemodialysis was applied since 23 postoperative days (POD). For a severe graft rejection on POD 23, 1.5 mg/kg of anti-thymocyte globulin (ATG) were given intravenously for three days. Abbreviation: LT, transplantation

**Figure 3:**
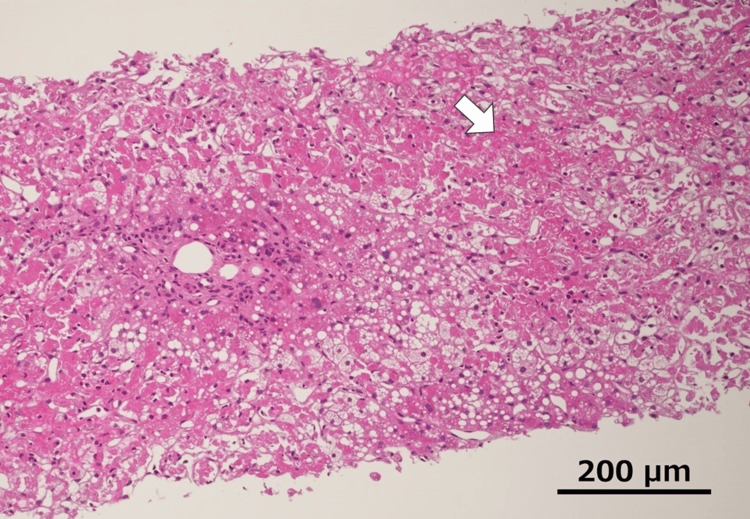
Histological findings of liver biopsy on POD 26 The histological finding (hematoxylin and eosin) of liver biopsy on POD 26. More than 90% of the hepatocytes had been destroyed and led to a centrilobular necrosis (arrow). More than 90% of the hepatocytes had been destroyed and led to a centrilobular necrosis (arrow). Abbreviation: POD, postoperative day

Postoperative immunosuppressive therapy consisted of tacrolimus (TAC), mycophenolate mofetil (MMF), and methylprednisolone (mPSL). Because of preoperative renal dysfunction, the target trough level of TAC was set at 8-10 ng/mL. The early postoperative course was uneventful in terms of surgical complications. However, due to persistent renal dysfunction requiring intermittent dialysis and careful fluid management, the patient remained hospitalized for close monitoring. On postoperative day (POD) 23, the patient suddenly developed severe acidemia (arterial pH 7.139), oliguria, markedly elevated hepatic enzymes (aspartate aminotransferase (AST) 7,994 IU/L, alanine aminotransferase (ALT) 3,960 IU/L), and coagulopathy (prothrombin time-international normalized ratio (PT-INR) 2.33) (Figure 2). Due to his critical condition, an immediate liver biopsy could not be performed. Acute graft rejection was suspected, and treatment with anti-thymocyte globulin (ATG; 1.5 mg/kg for three days) and plasma exchange was initiated. The TAC trough level was increased to 10-15 ng/mL. Severe renal failure eventually required repeated dialysis. A liver biopsy performed on POD 26 revealed that more than 90% of hepatocytes were destroyed, with centrilobular necrosis consistent with severe acute rejection (Figure 3).

After diagnosis, intensive multidisciplinary management was continued, including adjustment of immunosuppressive agents, continuous intravenous hydrocortisone infusion, plasma exchange, and strict infection control. The patient was managed collaboratively by the transplant surgery team, nephrology, and intensive care specialists, with additional nutritional and rehabilitation support provided throughout hospitalization. Liver function gradually improved over the following months: the total bilirubin (T-bil) level peaked at 29.6 mg/dL on POD 115 and then decreased to 1.3 mg/dL by POD 143. The graft dysfunction that began on POD 23 persisted but gradually improved with supportive treatment. However, during this period, the patient developed septic shock three times due to immunosuppression-associated infections, all of which were managed successfully with intensive care and antibiotic therapy. Despite these complications, no vascular or bleeding events occurred. However, renal dysfunction persisted despite conservative treatment, and maintenance hemodialysis was required. He has remained on regular hemodialysis three times a week, with a stable condition and no recurrence of hepatic dysfunction during follow-up. The patient was subsequently rehabilitated and returned to work while continuing regular dialysis (Figure 2). Both donor and recipient were negative for HBs-Ag and HCV-Ab. The patient was not immunized for hepatitis B prior to transplantation, but no post-transplant HBV infection was observed during follow-up. The patient has remained in stable condition for more than five years after transplantation, with no recurrence of hepatic dysfunction or other major complications.

## Discussion

Graft dysfunction is the most devastating complication, which mainly occurs during the early post-LT period [1,2]. It has a wide range of manifestations, including potentially reversible dysfunction and complete absence of function, termed primary nonfunction (PNF), delayed PNF, early allograft dysfunction (EAD), initial poor graft function, and posttransplant failure [2,3]. The cause and frequency of graft dysfunction depend on its definition. PNF is defined as graft failure immediately after reperfusion [4]. The Organ Procurement and Transplantation Network defines PNF as AST ≥ 3,000 IU/l and at least one of the following: PT-INR ≥ 2.5, arterial pH ≤ 7.30, venous pH ≤ 7.25, or lactate ≥ 4 mmol/l within seven days of LT [4]. The incidence rate was reportedly 0.9-7.2% [5], and it often leads to either re-LT or death of the patient.

EAD is defined as one or more of the following: a T-bil ≥ 10 mg/dl on POD 7, a PT-INR ≥ 1.6 on POD 7, or an ALT/AST ≥ 2000 IU/ml within POD 7 [2,6]. The risk factors for developing EAD include AST, ALT, T-bil, MELD score, red blood cell transfusion, thrombosis, and institutional experience [6,7].

The prevention and management of graft dysfunction rely on multiple perioperative strategies. Optimizing graft quality and graft-to-recipient size matching, ensuring adequate hepatic inflow and outflow, and minimizing cold and warm ischemia times are essential to reduce ischemia-reperfusion injury [8,9]. Early postoperative management focuses on strict hemodynamic control, maintenance of appropriate portal pressure, and prompt correction of coagulopathy. In addition, early screening for vascular and biliary complications using Doppler ultrasonography and CT enables timely interventions before irreversible hepatocyte injury occurs [8,9]. When acute rejection is suspected, rapid initiation or intensification of immunosuppressive therapy is crucial to prevent progression to graft failure [8,9].

Our case showed an AST of 7,994 IU/l, arterial pH of 7.139, and PT-INR of 2.33 on POD 23, which fulfilled the criteria for graft dysfunction except for the postoperative day. Furthermore, T-bil progressively increased to approximately 30 mg/dl. Histological findings showed destruction of more than 90% of the hepatocytes in the graft. There was a previous case report of graft dysfunction with 60% liver destruction due to rejection that did not respond to intensified immunosuppression or plasma exchange, resulting in re-LT [10]. It is likely that our case was highly lethal.

We considered registration on the DDLT waiting list for re-LT. However, due to the severe shortage of deceased donors in Japan, the indication for re-LT has been strictly limited [11]. Furthermore, the overall and graft survival rates for re-LT were reportedly low: the 1- and 5-year graft survival rates were 78% and 66%, respectively [11,12]. To identify patients who will recover from severe graft dysfunction by conservative therapy, future studies on biomarkers or scoring systems will be required.

Early graft failure is recognized as resulting from a complex interplay of clinical events such as AR, kidney failure, infection, and technical failure [1,13]. When early acute graft failure includes vascular complications, hepatic artery thrombosis (HAT, 4.8%) and PNF (4.3%) are the leading causes of graft failure [1,13]. In terms of graft dysfunction due to severe AR, all 6 (0.9%) of 90 cases displayed catastrophic outcomes with no recovery by conservative treatments [1,13]. A previous study in Japan demonstrated that the causes of early graft failure (<100 days post-LT) requiring re-LT were as follows: AR (38.7%), HAT, small-for-size syndrome, and PNF [10,14]. In addition, among the cases that underwent re-LT (n = 265) in Japan, AR (37.4%) was the leading cause of graft failure [3,14].

In Japan, living donor LT is the mainstay, in which liver regeneration is crucial for successful LT. Clinical events in the early post-LT period, such as AR, critically interfere with the growth of a partial liver graft [15]. In our DDLT case, a sufficient liver volume was relatively tolerated AR compared with living donor LT cases. However, severe AR destroyed almost all hepatocytes, leading to graft dysfunction.

A higher mortality rate in the first year post-LT was reported in recipients with pre-LT renal dysfunction compared to those without [11,16]. Meanwhile, a previous study (n = 174) reported that pre-LT serum creatinine (sCre) level was negatively correlated with AR development, that is, lower sCre levels were significantly associated with AR occurrence [12,17]. The study demonstrated that patients with an sCre higher than 2.7 mg/dl at transplantation had a lower risk of acute AR [17]. Furthermore, renal dysfunction post-LT was associated with severe infection resulting in a poor prognosis [13,18]. Thus, renal dysfunction may be associated with weakened immunity, suggesting that the doses of immunosuppressants should be reduced. Indeed, minimizing immunosuppression reportedly prevents renal dysfunction due to the nephrotoxic effects of CNI [14,19]. Recipients with pre-LT sCre levels ≥ 1.2 mg/dl or baseline GFR ≤ 70 ml/min/1.73 m² were reported to have a three- and 12-fold higher risk of developing permanent renal dysfunction after LT, respectively [14,19].

Accordingly, we set 8-10 ng/ml as the TAC blood trough level in this case, given the sCre of 1.72 mg/dl at pre-LT, which was a relatively lower concentration of TAC than in patients without renal impairment. It potentially led to severe AR and graft dysfunction, conversely.

To achieve both renal preservation and prevention of AR, delayed CNI introduction combined with induction therapy (daclizumab, basiliximab, or ATG) has reportedly been applied [15-17]. A European multicenter randomized trial of 525 LT recipients with normal renal function compared standard-dose TAC (target >10 ng/ml), reduced-dose TAC (≤8 ng/ml with MMF), and delayed reduced-dose TAC with daclizumab induction; the delayed group showed less GFR reduction and a lower incidence of AR [15]. Another European study of 990 LT recipients compared conventional TAC dosing (10-15 ng/ml) with reduced-dose delayed induction (basiliximab, 5-8 ng/ml) and demonstrated lower renal damage and improved graft survival in the delayed group [16]. A Spanish study of 40 recipients with pre-transplant renal dysfunction found that low-dose ATG induction therapy provided similar renal protection as basiliximab induction therapy [17].

These studies suggest that delayed treatment with reduced-dose TAC combined with anti-CD25 antibody or ATG induction therapy is a promising strategy for renal protection and may also suppress acute rejection, potentially reducing the risk of graft failure [17]. Although these treatments are not yet covered by insurance in Japan, they could have been suitable for our case. Given the increasing complexity of post-LT management, incorporating such individualized immunosuppressive approaches may help refine future treatment strategies, particularly for recipients with impaired renal function.

## Conclusions

Our rare case achieved recovery from more than 90% hepatocyte loss due to severe acute rejection through conservative treatment alone. This outcome demonstrates that even in cases of extreme graft dysfunction, careful monitoring, appropriate immunosuppressant adjustment, and comprehensive supportive therapy can result in favorable recovery. Recognition of clinical parameters that predict liver graft recovery may help guide future treatment strategies and clinical decision-making in similar high-risk liver transplant cases.
